# miR-449a inhibits colorectal cancer progression by targeting SATB2

**DOI:** 10.18632/oncotarget.10900

**Published:** 2016-07-28

**Authors:** Xiaohua Sun, Sanhong Liu, Pengfei Chen, Da Fu, Yingyong Hou, Jin Hu, Zhi Liu, Yuhang Jiang, Xinwei Cao, Chunyan Cheng, Xi Chen, Yu Tao, Cuifeng Li, Yiming Hu, Zhanjie Liu, Yu Zhan, Jie Mao, Qi Wang, Yushui Ma, Xianling Cong, Ran Sun, Yufang Shi, Mingliang Wang, Xiaoren Zhang

**Affiliations:** ^1^ Key Laboratory of Stem Cell Biology, Institute of Health Sciences, Shanghai Institutes for Biological Sciences, Chinese Academy of Sciences and Shanghai Jiao Tong University School of Medicine, Shanghai, 200031, China; ^2^ Collaborative Innovation Center of System Biomedicine, Shanghai Jiao Tong University School of Medicine, Shanghai, 200240, China; ^3^ Institute of Health Sciences, Shanghai Institutes for Biological Sciences, Chinese Academy of Sciences and Shanghai Jiao Tong University School of Medicine, Shanghai, 200031, China; ^4^ Department of Pathology, Zhongshan Hospital, Fudan University, Shanghai, 200032, China; ^5^ General Surgery Department, Ruijin Hospital, Shanghai Jiao Tong University School of Medicine, Shanghai, 200025, China; ^6^ Department of Nuclear Medicine, Shanghai 10th People's Hospital, Tongji University School of Medicine, Shanghai, 200072, China; ^7^ Tissue Bank, Scientific Research Center, China-Japan Union Hospital, Changchun, 130033, China; ^8^ Present address: Shanghai Institute for Advanced Immunochemical Studies, ShanghaiTech University, Shanghai, 201210, China

**Keywords:** colorectal cancer, miR-449a, SATB2, tumorigenesis

## Abstract

miR-449a has been reported to act as a tumor suppressor in several cancers, however, it is controversial whether it inhibits tumor growth in colorectal cancer. The mechanisms underlying its expression and functions in colorectal cancers are still largely unknown. SATB2 is a sensitive and specific marker for CRC diagnosis. However, the mechanisms by which the expression and functions of SATB2 are regulated still remain to be clarified. We investigated the expression and functional significance of miR-449a and SATB2 and the mechanisms of their dysregulation in human CRC cells. miR-449a overexpression or SATB2 depletion inhibited tumor growth and promoted apoptosis in colorectal tumor cells *in vitro* and in xenograft mouse model, partially by downregulating SATB2. Expression of miR-449a was increased epigenetically via knocking down their targets, particularly SATB2. miR-449a was downregulated and STAB2 expression was upregulated in human CRCs. Their expressions were significantly associated with overall survival of CRC patients. Our findings demonstrate the existence of a miR-449a-SATB2 negative feedback loop that maintains low levels of miR-449a as well as high level of SATB2, thereby promoting CRC development.

## INTRODUCTION

Colorectal cancer (CRC) is ranked the third most common cancer and the fourth leading cause of death among all human cancers worldwide, with 1.2 million cases identified every year [1, 2]. As the 5-year survival rate and treatment efficiency correlate with the stage at which the cancer is detected [3], identifying novel biomarkers for CRC early diagnosis and therapy is urgently needed.

MicroRNAs (miRNAs) are a class of highly conserved short (approximately 23 nt) single-stranded RNAs that bind to the 3′ untranslated region (UTR) of target messenger RNA (mRNA) and mediate mRNA degradation or translational repression [4]. Thus far, miRNAs have been found to be involved in a series of cellular processes including apoptosis, cancer development, metastasis and differentiation [5–7]. Some miRNAs function as oncogenes or tumor suppressors and are important in cancer development. For instance, miR-449a has been reported to act as a tumor suppressor, and its expression is downregulated in several cancers including ovarian, lung, bladder, prostate and gastric cancers [8–10]. miR-449a induces cell cycle arrest at G1 phase by directly targeting CDK6 and CDC25A, which leads to the inhibition of pRb-E2F1 activity in prostate cancer cells and breast cancer cells [11–14], and directly targets Bcl-2, HDAC1 and Sirt1 to promote cell death. However, the mechanism underlying the regulation of decreased miR-449a expression and tumor suppression in CRC remains largely unknown.

Special AT-rich sequence-binding protein 2 (SATB2), which is known as a nuclear matrix-associated transcription factor and epigenetic regulator, is an evolutionarily conserved transcription factor that plays crucial roles in osteoblast differentiation, neuronal development and craniofacial patterning [15–17]. SATB2 is also a sensitive and highly specific marker for CRC, functioning as a diagnostic marker that clinically distinguishes CRC from other types of cancer [18–22]. However, the function of SATB2 in CRC development is poorly understood.

In this study, we report that miR-449a directly targets SATB2. SATB2 knockdown or miR-449a overexpression inhibits CRC cell growth *in vitro* and in xenograft mouse model. miR-449a level is decreased, leading to increased SATB2, which subsequently downregulates miR-449a in human CRC. Furthermore, reduced miR-449a level is attributable to DNA hypomethylation and histone hypoacetylation in human CRC. These findings reveal a negative feedback loop for the suppression of CRC development. Taken together, our data indicate that this miR-449a-SATB2-mediated feedback loop plays critical roles in human CRC development and that the components of this feedback loop may serve as potential targets for the diagnosis and treatment of human CRC.

## RESULTS

### miR-449a expression is reduced in human CRC

**Figure 1:**
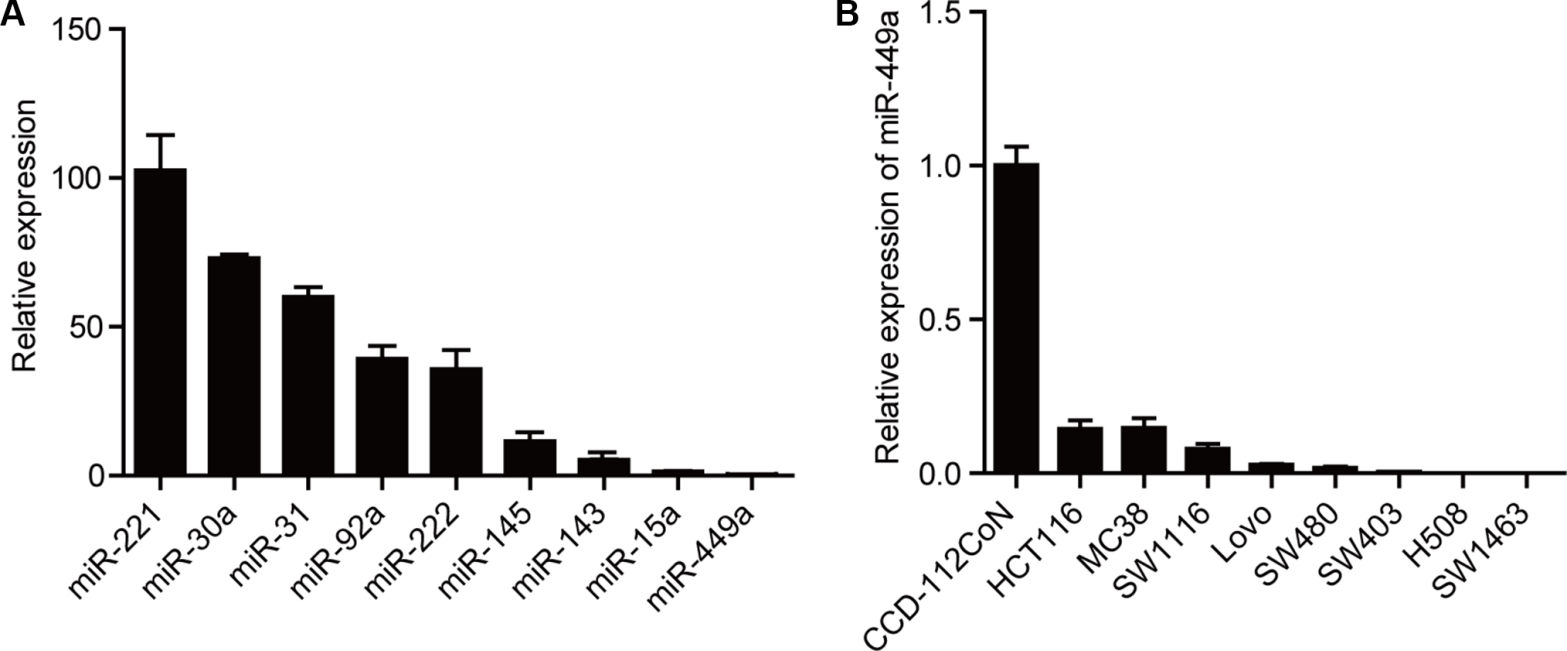
miR-449a expression is lower in CRC compared to other miRNAs (**A**) The mRNA expression levels of different miRNAs in CT26 cells. U6 was used as control. (**B**) The mRNA level of miR-449a in different CRC cell lines. The normal colon epithelial cell line CCD-112CoN was used as a control.

With the goal of identifying dysregulated miRNAs in CRC cells, we examined CT26 cells for levels of several miRNAs that are aberrantly expressed in other cancer types. Among these miRNAs, miR-449a was found to exhibit much lower expression, even compared with miR-143/145, two putative tumor suppressive microRNAs (Figure 1A) [23, 24]. Furthermore, the miR-449a level was found to be significantly decreased in a number of CRC cell lines compared with the CCD-112CoN cell line, a non-tumorigenic colon epithelial cell line (Figure 1B). Taken together, these results suggest that decreased miR-449a expression may correlate with the progression of CRC.

### miR-449a overexpression suppresses CRC cell growth and promotes apoptosis *in vitro* and *in vivo*

**Figure 2:**
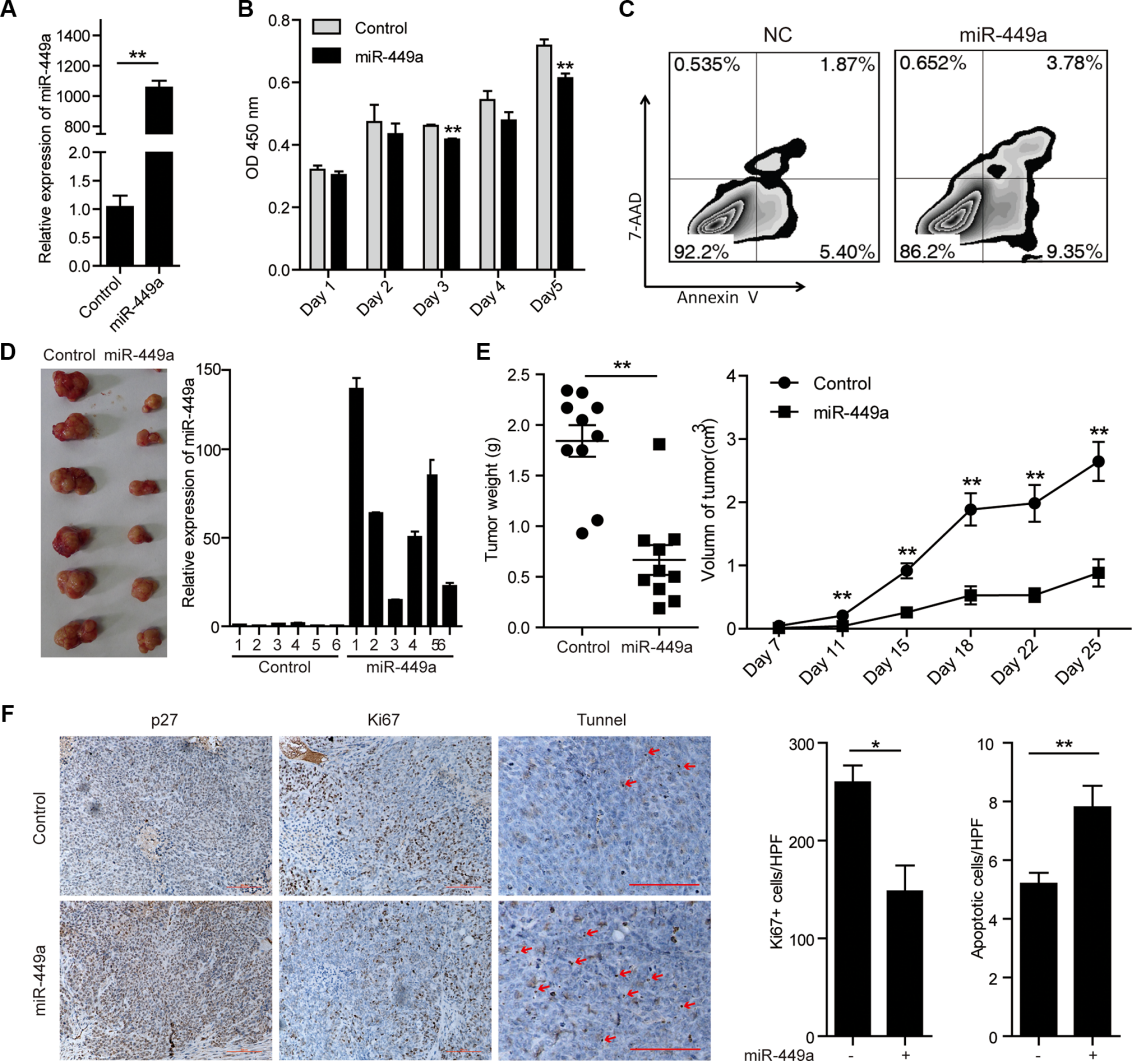
Introduction of miR-449a suppresses CRC cell growth and promotes apoptosis *in vitro* and *in vivo* (**A**) The mRNA level of miR-449a in HCT116 cells stably expressing the negative control or miR-449a. (**B**) MTT results of HCT116 cells stably expressing miR-449a or the negative control on the indicated days. (**C**) Annexin V/7-AAD flow cytometry results of HCT116 cells stably expressing miR-449a or the negative control. (**D**) Tumor growth in mice injected with HCT116 cells stably expressing miR-449a or the negative control. (**E**) Tumor weight (left) and volume (right) in mice injected with HCT116 cells stably expressing miR-449a or the negative control (*n* = 10). (**F**) Immunohistochemistry results for p27 and Ki67. TUNEL assays of tumors derived from HCT116 cells stably expressing miR-449a or the negative control.

To address the physiological effect of miR-449a on CRC cell growth, we constructed HCT116 cells stably expressing miR-449a (Figure 2A). miR-449a overexpression significantly suppressed cell growth and induced apoptosis *in vitro* (Figure 2B–2C). Consistently, miR-449a overexpression in CRC cells led to reduced tumor volume and weight, fewer Ki67-positive cells and more TUNEL-positive cells in tumors (Figure 2D–2F). In addition, miR-449a elevated the expression of the cell cycle inhibitory protein p27 and reduced the expression of cyclin D1 and Bcl-2 in CRC cells (Figure 2F and Supplementary Figure S1).

### SATB2 is a direct target of miR-449a in human CRC

**Figure 3:**
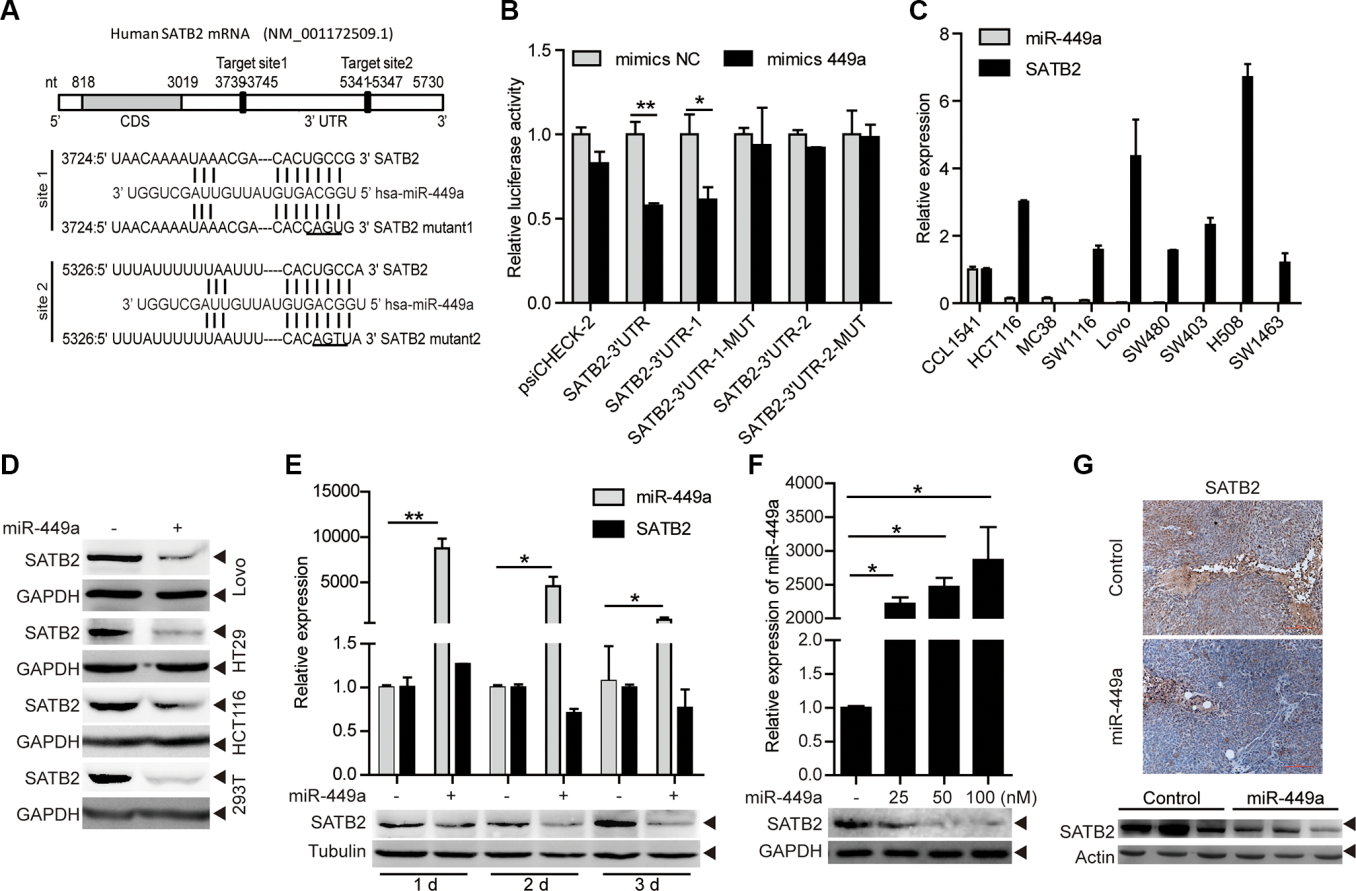
SATB2 is a direct target of miR-449a (**A**) The predicted binding sites of miR-449a in the SATB2 3′UTR. (**B**) Luciferase activity of the SATB2 3′UTR reporter in 293T cells transfected with 100 nmol/L miR-NC or miR-449a mimics, 200 ng of the psiCHECK-SATB2 3′UTR reporter plasmid, SATB2-3′UTR-1 (binding site 1), SATB2-3′UTR-1-MUT (binding site 1 mutant), SATB2-3′UTR-2 (binding site 2), SATB2-3′UTR-2-MUT (binding site 2 mutant) or the control reporter plasmid for 24 hours. (**C**) miR-449a, SATB2 mRNA expression levels in different CRC cell lines using the CCD-112CoN cell line as the control. (**D**) SATB2 protein levels in Lovo, HT29, HCT116 and 293T cells treated with 100 nmol/L miR-NC or miR-449a mimics. (**E**) Effect of time-dependent inhibition of miR-449a mimics (upper panels) on the SATB2 protein (lower panels) in HCT116 cells. (**F**) Effect of dose-dependent inhibition of miR-449a mimics (upper panels) on the SATB2 protein (lower panels) in HCT116 cells. (**G**) Immunohistochemistry results (upper panels) and western blotting results for SATB2 in xenograft tumors derived from HCT116 cells stably expressing miR-449a or the negative control.

miR-449a overexpression in CRC cell line DLD1 cells does not inhibit pRb phosphorylation and E2F1 expression, despite a reduction in CDK6 and CDC25A protein expression, suggesting that other targets of miR-449a may contribute to its function [13]. To evaluate potential targets of miR-449a in CRC, we used online search tools (Target Scan, miRanda) to identify putative targets. Among many potential targets, SATB2 is known as a nuclear matrix-associated transcription factor and epigenetic regulator and serves as a sensitive and highly specific marker for CRC [18–20]. The 3′UTR of the SATB2 mRNA has 2 predictive target sites with high complementarity to miR-449a (Figure 3A). To determine whether SATB2 is a direct target of miR-449a, we separately cloned the entire sequence and the 2 binding sites into the 3′UTR of a luciferase reporter vector (SATB2-3′UTR, SATB2-3′UTR-1, SATB2-3′UTR-2). Co-transfection with miR-449a mimics, but not the nonspecific control miRNA (NC), specifically decreased luciferase activity, whereas both point mutations (SATB2-3′UTR-1-MUT, SATB2-3′UTR-2-MUT) rescued the miR-449a-mediated repression of luciferase levels, indicating that miR-449a can directly target SATB2, particularly by site 1 (Figure 3B). We also performed the same assay in HCT116 and RKO cells, the results were similar to that found in 293T cells (data not shown). In addition, the SATB2 mRNA level was elevated and the miR-449a mRNA level decreased in different CRC cell lines, revealing a negative correlation between miR-449a and SATB2 (Figure 3C, the expression of miR-449a has shown in Figure 1B). We also found that miR-449a mimics markedly decreased the protein level of SATB2 in a time- and dose-dependent manner (Figure 3D–3F). Immunohistochemical analysis and western blotting showed that miR-449a overexpression dramatically inhibited SATB2 in xenografted tumors (Figure 3G).

### SATB2-specific knockdown and miR-449a overexpression elicit similar phenotypes *in vitro* and *in vivo*

**Figure 4:**
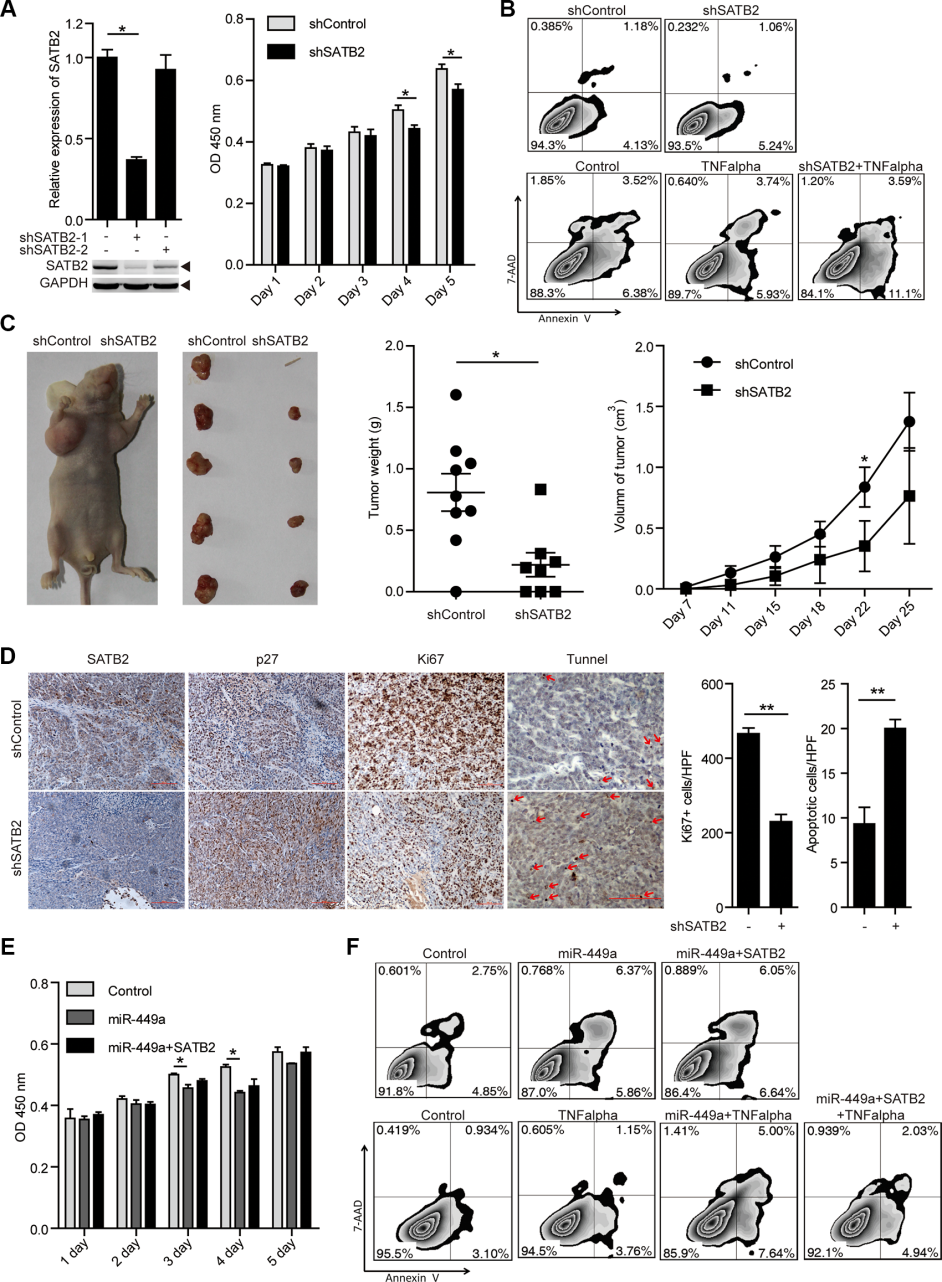
SATB2 knockdown inhibits CRC cell growth *in vitro* and *in vivo* (**A**) SATB2 mRNA (upper panels on the left) and protein (lower panels on the left) levels in HCT116 cells stably expressing the negative control, shSATB2-1 or shSATB2-2 (2 separate sequences to knockdown SATB2). MTT results of HCT116 cells stably expressing shSATB2 or the negative control on the indicated days (right). (**B**) Annexin V/7-AAD flow cytometry results of HCT116 cells stably expressing shSATB2 or the negative control with or without TNF alpha (10 ng/ml). (**C**) Tumor growth in mice injected with HCT116 cells stably expressing shSATB2 or the negative control (left 2 panels). Tumor weight (middle) and volume (right) in mice injected with HCT116 cells stably expressing shSATB2 or the negative control (*n* = 9). (**D**) Immunohistochemistry results of SATB2, p27, and Ki67. TUNEL assays in tumors derived from HCT116 cells stably expressing shSATB2 or the negative control. (**E** )MTT results of HCT116 cells stably expressing miR-449a or the negative control with or without SATB2-CDS overexpression on the indicated days. (**F**) Annexin V/7-AAD flow cytometry results of HCT116 cells stably expressing miR-449a or negative control with or without SATB2-CDS overexpression and TNF alpha (10 ng/ml).

To investigate the roles of SATB2 in tumor cell growth and survival, we treated HCT116 cells with 2 different shRNAs specific for SATB2 (shSATB2-1, shSATB2-2). With a better effect on knocking down SATB2, we used shSATB2-1 for following experiments. Western blot analysis confirmed the specific knockdown of SATB2 (Figure 4A). We found that SATB2 knockdown inhibited cell growth and enhanced sensitivity to TNF-induced apoptosis; however, knocking down SATB2 alone had little effect on apoptosis *in vitro* without proapoptotic reagents (Figure 4A–4B). Furthermore, reduced tumor volumes and weights, fewer Ki67-positive cells and more TUNEL-positive cells were also observed in the xenografted tumors of SATB2-knockdown cells, suggesting that the growth arrest induced by miR-449a is similar with SATB2 (Figure 4C–4D).

As shown in Figure 4D, a profound induction in p27 levels was observed after shSATB2 treatment. To determine whether miR-449a mediates its growth-suppressive effects primarily by downregulating SATB2, we overexpressed SATB2 in tumor cells overexpressing miR-449a. We noted that SATB2 overexpression partially rescued the effects of miR-449a on cell growth and apoptosis induced by TNF. The above evidence indicates that the growth-repressing effects of miR-449a are in part facilitated by SATB2 downregulation (Figure 4E–4F).

### miR-449a is epigenetically repressed and activated by epigenetic drugs and SATB2 knockdown

As tumor suppressors, inhibitors of DNA methylation and histone deacetylase (HDAC) have been reported to induce the expression of miRNAs [25, 26]. Therefore, we hypothesized that the downregulation of miR-449a might be attributable to the aberrant epigenetic events that occur in CRC.

**Figure 5:**
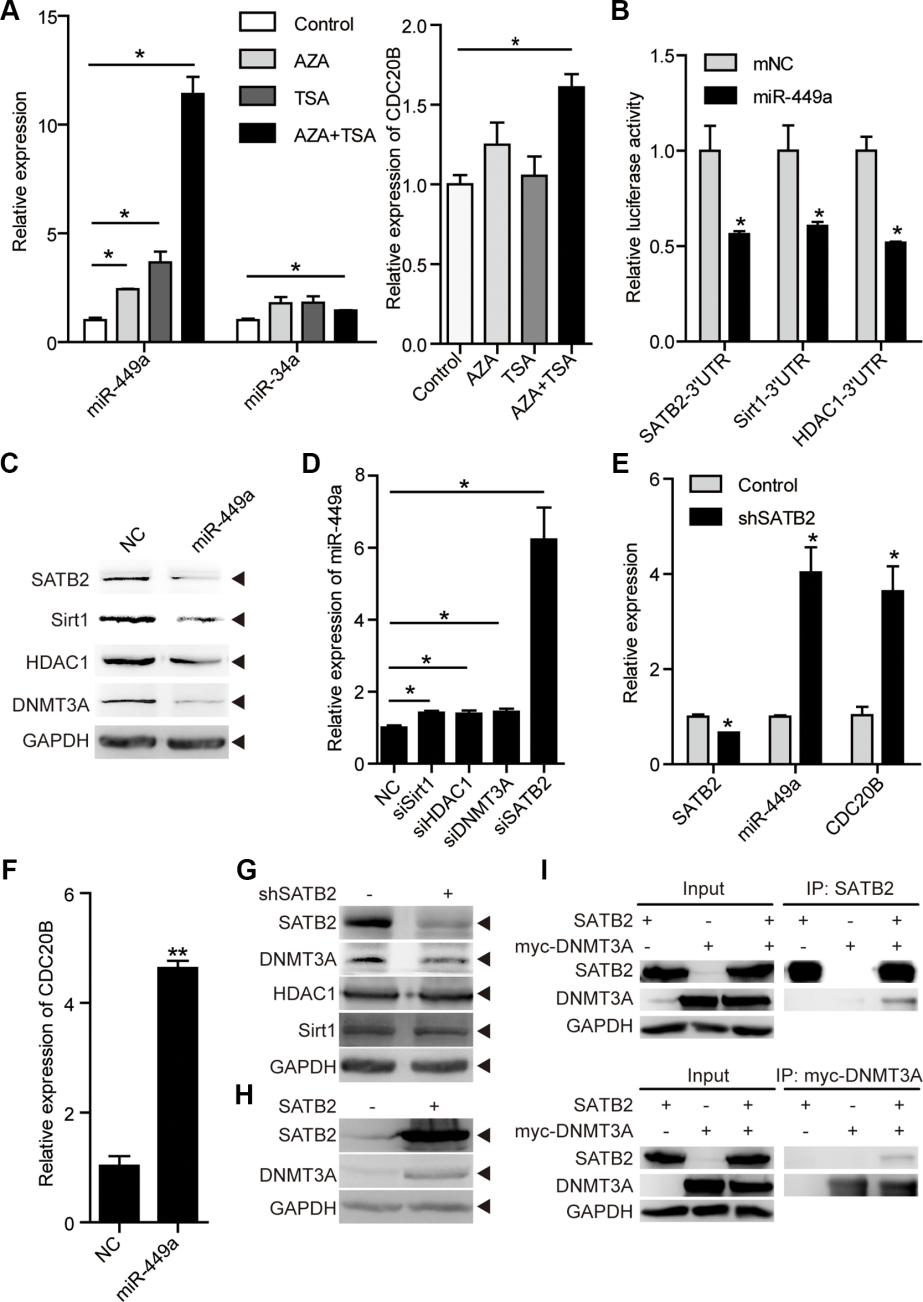
miR-449a is activated by epigenetic drugs and SATB2 knockdown (**A**) miR-449a and miR-34a mRNA levels after AZA and TSA treatment (left). The CDC20B mRNA level after AZA and TSA treatment (right) in RKO cells. (**B**) Luciferase activity of the SATB2, HDAC1, and Sirt1 3′UTR reporters in 293T cells transfected with 100 nmol/L miR-NC or miR-449a mimics and 200 ng of reporter plasmid for 24 hours (left). (**C**) SATB2, Sirt1, HDAC1, and DNMT3A protein levels in HCT116 cells treated with 100 nmol/L miR-NC or miR-449a mimics (right). (**D**) miR-449a mRNA levels after transfection of HCT116 cells with siSirt1, siHDAC1, siDNMT3A or siSATB2. (**E**) SATB2, miR-449a, and CDC20B mRNA levels after SATB2 knockdown in HCT116 cells. (**F**) miR-449a and CDC20B mRNA levels after transfection of HCT116 cells with miR-449a mimics. (**G**) DNMT3A, Sirt1, HDAC1 protein levels after SABT2 knockdown. (**H**) DNMT3A protein level after SABT2 overexpression. (**I**) Co-immunoprecipitation (Co-IP) of SATB2 and myc-tagged-DNMT3A (myc-DNMT3A) expressed in HCT116 cells, cell lysates were immunopricipitated with the indicated antibodies.

The small-molecule DNA methylation inhibitor AZA or HDAC inhibitor TSA have been shown to effectively reverse gene repression, and this combination appears to influence pathways in cancer and apoptosis (Supplementary Figures S2 and S3) [27–29]. Indeed, the combination of AZA and TSA dramatically induced apoptosis and significantly increased miR-449a mRNA levels, whereas AZA or TSA treatment alone only slightly increased the miR-449a level (Figure 5A and Supplementary Figure S4A). In contrast, miR-34a was not induced by this treatment, although CDC20B, the host gene of miR-449a, was induced by combined AZA and TSA treatment.

We then tested whether miR-449a regulates the expression of DNA methyltransferase 3A (DNMT3A), histone deacetylase 1 (HDAC1), NAD+-dependent protein deacetylase Sirt1 and SATB2, which are widely studied as epigenetic regulators, and some of which are miR-449a targets (Figure 5B and 5C) [11, 30–32]. We examined whether these molecules would influence miR-449a levels by transiently transfecting HCT116 cells with specific siRNAs against Sirt1, HDAC1, DNMT3A or SATB2. SATB2 knockdown increased miR-449a dramatically, whereas siSirt1, siHDAC1 or siDNMT3A exhibited only slight effects (Figure 5D). Simultaneously, SATB2 knockdown induced the expression of CDC20B, which is probably regulated by miR-449a (Figure 5E–5F and Supplementary Figure S4B).

We next addressed whether SATB2 had an effect on these epigenetic regulators. SATB2 knockdown decreased the level of DNMT3A protein but not of Sirt1 or HDAC1, whereas SATB2 overexpression increased the level of DNMT3A protein (Figure 5G and 5H). Next, we co-transferred expression vectors encoding SATB2 and DNMT3A into HCT116 cells and immunoprecipitated the lysates with antibodies directed against either protein. Immunoblots reveals that SATB2 was present in DNMT3A immunoprecipitates and *vice versa* (Figure 5I). This miR-449a-mediated decrease in DNMT3A protein levels suggests that miR-449a indirectly regulates DNMT3A through SATB2 (Figure 5C).

Taken together, these results indicate that miR-449a is epigenetically repressed and activated by epigenetic drugs and its targets, forming a negative feedback loop to regulate its expression.

### Mutual regulation between miR-449a and miR-34a in CRC

**Figure 6:**
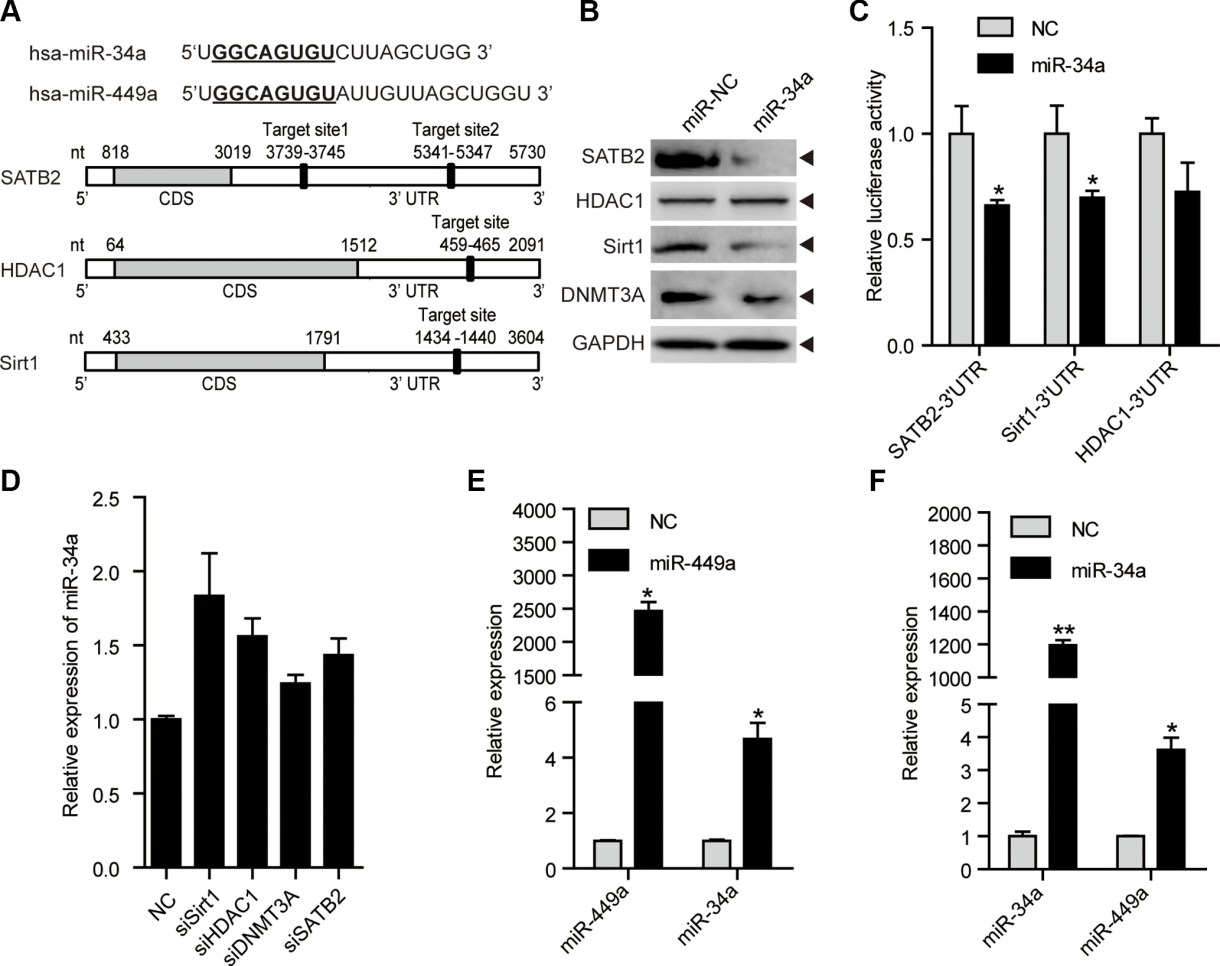
miR-34a shares similar targets with miR-449a, and they can induce each other mutually (**A**) Schematic depiction of miR-34a and miR-449a and matching targets, namely, SATB2, HDAC1, and Sirt1. (**B**) SATB2, HDAC1, Sirt1 and DNMT3A protein levels after treatment of HCT116 cells with 100 nmol/L miR-NC or miR-34a mimics. (**C**) Luciferase activity of the SATB2, HDAC1, and Sirt1 3′UTR reporters in 293T cells transfected with 100 nmol/L miR-NC or miR-34a mimics and 200 ng of reporter plasmid for 24 hours. (**D**) miR-34a mRNA levels after transfection with siSirt1, siHDAC1, siDNMT3A or siSATB2. (**E**) miR-449a mRNA levels after treatment of HCT116 cells with 100 nmol/L miR-NC or miR-34a mimics. (**F**) miR-34a mRNA levels after treatment of HCT116 cells with 100 nmol/L miR-NC or miR-449a mimics.

miR-34a, a putative tumor suppressor miRNA that has been shown to repress tumor growth and metastasis, shares identical seed sequences and target genes with miR-449a, such as SATB2, Sirt1 and HDAC1 [33–38]. We found that miR-34a also targets Sirt1, SATB2 and HDAC1 in CRC cells (Figure 6A–6C). Similar to miR-449a, miR-34a reduced the DNMT3a protein level possibly because miR-34a downregulated SATB2 (Figure 6B). Depletion of Sirt1, HDAC1, DNMT3A or SATB2 slightly increased the miR-34a mRNA level (Figure 6D).

miR-449a has been reported to induce apoptosis potently and upregulate p53 activity, followed by miR-34a induction [31]. We hypothesized that miR-449a could induce miR-34a expression. We found that miR-449a increased miR-34a expression (Figure 6E). We also detected that miR-34a induced miR-449a expression, which further supplemented the model raised by Lize (Figure 6F) [31]. These data indicate that miR-34a targets identical downstream genes including SATB2, Sirt1, HDAC1 and DNMT3a, which subsequently upregulate the expression of miR-449a and 34a. Thus, decreased miR-34a expression contributes to a miR-449a-mediated negative feedback loop to maintain low levels of miR-449a/34a and high levels of SATB2, Sirt1, HDAC1 and DNMT3a in colorectal cancer cells.

### Reduction of miR-449a during tumorigenesis is associated with survival

**Figure 7:**
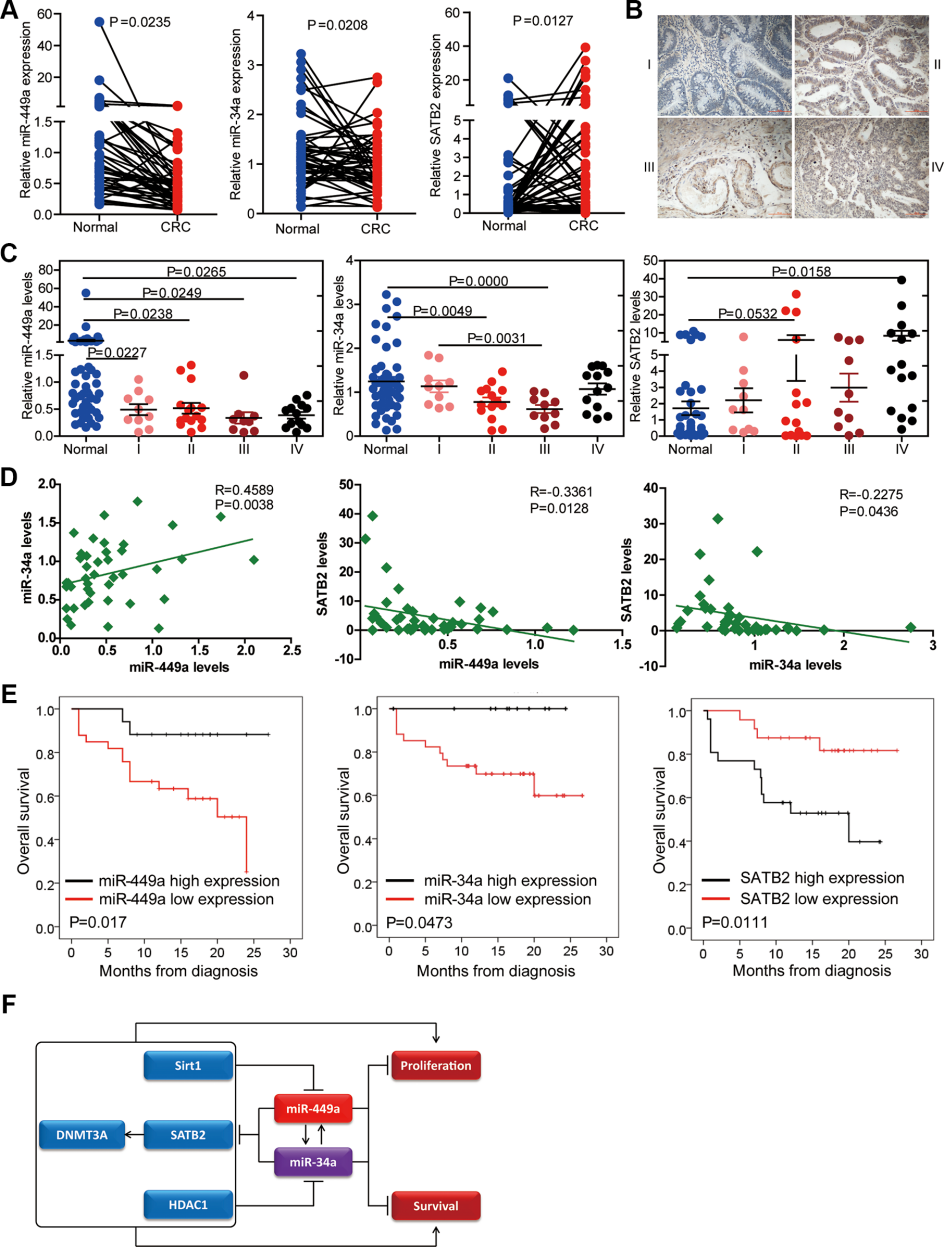
Relevance of the miR-34a/miR-449a-SATB2 pathway in human CRC development and progression (**A**) Assessment of the miR-449a, miR-34a, and SATB2 levels *via* real-time PCR analysis of total RNA from 50 paired normal and CRC colon tissues. (**B**) Representative immunohistochemical images of SATB2 staining according to tumor stage. (**C**) Assessment of the miR-449a, miR-34a, and SATB2 levels *via* real-time PCR analysis of total RNA from 50 paired normal and CRC colon tissues according to tumor stage. (**D**) Correlation between the expression levels of miR-449a and miR-34a and SATB2 in the clinicl samples described. Each data point represents an individual colon tissue sample, and the correlation coefficient (R) is shown. (**E**) Kaplan-Meier estimates of overall survival in 50 patients with CRC. (**F**)Schematic representation of the proposed roles of the miR-34a/miR-449a-SATB2 feedback loop in human CRC development and progression.

To investigate the clinical relevance of the expression of miR-449a, miR-34a and their target gene SATB2, we detected their expression levels by qPCR in 50 paired human colorectal normal and cancer tissues which included 10 stage I, 15 stage II, 10 stage III and 15 stage IV samples, respectively (Supplementary Table S2). We found that miR-449a and miR-34a levels were decreased in cancer tissues compared with normal tissues, although their expressions were comparable among different stages of CRC according to the TNM grading score (Figure 7A and 7C). In contrast, SATB2 mRNA levels were higher in CRC tissues than in normal tissues (Figure 7A). Representative SATB2 staining gradually increased during CRC progression (Figure 7B). miR-449a expression was positively correlated with miR-34a expression in these patients, while miR-449a and miR-34a were negatively correlated with SATB2 expression (Figure 7D). We also determined the relevance of the expression of miR-449a/34a and SATB2 on the overall survival of patients with CRC. Kaplan-Meier analysis showed a significant impairment of overall survival with decreasing miR-449a/34a expression or increasing SATB2 expression (Figure 7E).

## DISCUSSION

Dysregulation of miRNA genes has been reported to influence human tumorigenesis. miR-449a has been shown to suppress cell proliferation, reduce cell survival and/or inhibit migration and invasion by targeting various genes in many cancer types [8–10, 39–41]. However, the regulation of miR-449a expression and its roles in CRC are largely unknown. Here, we report the existence of a negative feedback loop that maintains reduced levels of miR-449a and miR-34a and that directly promotes CRC cell growth and survival through upregulating a novel target: SATB2.

Although miR-449a expression was reported to increase in 24 patients with CRC, our data revealed reduced expression of miR-449a and miR-34a in 50 CRC tissue samples compared with paired colorectal normal tissues [42]. Indeed, miR-449a has been shown to be downregulated in several cancers, although the mechanism underlying its reduction remains largely unknown. DNA methylation and histone deacetylation are important epigenetic modifications responsible for transcriptional silencing. We found that miR-449a can also be induced by AZA and TSA in CRC cells and further identified that that knockdown of miR-449a targets, namely, Sirt1, HDAC1, and particularly SATB2, upregulates miR-449a levels. Thus, DNA methylation *via* DNMT3a, stabilized by SATB2, integrates with histone acetylation mediated by HDAC1 and Sirt1 to contribute to the decreased expression of miR-449a and miR-34a, forming a negative feedback loop.

miR-449a has been reported to suppress tumor cell proliferation/growth and survival by targeting CDK6, CDC25A, Sirt1, HDAC1, cyclinD1, WISP2, and c-Met [11–14]. Here, we identify the suppressive effects of miR-449a on CRC cell growth and survival, which occur at least partially if not completely through SATB2. Firstly, we verified that SATB2 is a direct target gene of miR-449a. Secondly, we found that miR-449a overexpression suppressed tumor growth and induced apoptosis *in vitro* and *in vivo* in CRC cells. Similarly, SATB2 knockdown exhibited suppressive effects on CRC cell growth and survival *in vitro* and particularly in xenograft mouse model. Thirdly, SATB2 overexpression partially rescued the growth suppression and apoptosis induced by miR-449a overexpression. Meanwhile, we discovered that SATB2 knockdown could upregulate CDC20B expression, the host gene of miR-449a. Given to that miR-449a could repress SATB2 expression, we hypothesized that miR-449a regulated its host gene CDC20B indirectly by downregulating SATB2. Thus, SATB2 functions as a critical mediator in both the negative feedback loop that maintains low levels of miR-449a and miR-34a and the suppression of tumor growth and survival.

SATB2 has been shown to be a target of miR-31 and miR-182, and this protein is associated with metastasis and poor prognosis in CRC [43–45]. However, we found that SATB2 knockdown significantly suppressed tumor proliferation in xenograft mouse model, suggesting that SATB2 upregulation constitutes an early step in colorectal tumorigenesis and that it plays different roles in tumor progression. Indeed, the tumor cell growth-repressing effects of miR-449a are in part facilitated *via* the downregulation of SATB2, indicating the existence of other targets that impact tumor growth and that are regulated by miR-449a. In addition, we identified that SATB2 can bind to DNMT3A and upregulate its protein expression, thereby indirectly affecting tumor growth and miR-449a and miR-34a expression *via* DNA methylation. SATB2 inhibited tumor growth indirectly via DNMT3A and miR-449a/34a expression. Certainly, STAB2 may function through other mechanisms including augmenting deltaNp63 that need to be further identified [46].

miR-34a is a putative tumor suppressor miRNA and shares identical seed sequences with miR-449a. In accordance with their similarity, miR-34a shares the same targets as miR-449a, such as SATB2, HDAC1 and Sirt1. We found that the two miRNAs respond to each other: miR-34a overexpression induces miR-449a and *vice versa*. These findings provide new clues for the downregulation of miR-449a and miR-34a in CRC. Furthermore, the identical seed sequences and the association between miR-449a and miR-34a ensure that the miR-449a/34a-SATB2-mediated negative feedback loop plays a critical role in CRC development (see model in Figure 7F). A similar tendency that high levels of miR-449a and miR-34a are associated with prolonged overall survival in CRC was also demonstrated, indicating the importance of this negative feedback loop in CRC development.

In the normal colon, SATB2 expression is restricted to the glandular cells lining the lower gastrointestinal tract, whereas SATB2 expression is completely lacking in endothelial cells and peripheral nerves [18]. SATB2 has been shown to be increased or decreased in CRC, although with no mention of which cell type overexpresses this factor, revealing the importance of the section used in IHC [18]. For example, SATB2 expression in a section with more glandular cells may be much higher than in sections with fewer glandular cells and more endothelial cells, regardless of the stage of the section. This possibility may explain the reduced expression of SATB2 in poorly differentiated CRCs compared with well-differentiated and moderately differentiated primary tumors in some studies. We examined the expression of SATB2 mRNA and miR-449a/34a in 50 paired CRC and normal tissues. We observed the increased expression of STAB2 in CRC tissues compared to normal tissues. Consistently, increased SATB2 expression was negatively correlated with reduced miR-449a or miR-34a expression in CRC. Importantly, our clinical data indicated that low levels of miR-449a/34a and high level of SATB2 were significantly correlated with impaired overall survival in CRC patients.

In summary, our results indicate the existence of a negative feedback loop that maintains low levels of miR-449a and miR-34a as well as high level of SATB2 to promote colorectal tumor development. Our findings also reveal that miR-449a can serve as a promising novel target for the diagnosis and treatment of human CRC.

## MATERIALS AND METHODS

### Primary tissue

Our analysis was performed based on tumor specimens from 50 patients with CRC, which were obtained from Shanghai 10th People's Hospital for diagnostic purposes. The tumor specimens from these patients were analyzed by mRNA expression and immunohistochemistry (IHC). The tumors were obtained with permission from the local ethics committee.

### Animal experiments

Four-week-old C57BL/6 male mice and BALB/c nude mice were purchased from Shanghai Laboratory Animal Center, Chinese Academy of Sciences, Shanghai. All experiments were performed under the guidelines for the care and use of laboratory animals.

In total, 2 × 10^6^ HCT116 cancer cells expressing pLVX-miR-449a (miR-449a overexpression) or pLVX-shSATB2 (SATB2 knockdown) were injected subcutaneously into the nude mice. The tumor size was measured twice every week. After 4 weeks, the mice were euthanized by cervical dislocation, and the tumors were dissected. The tumor weight was measured, and the tumor volume was calculated using the following formula: D × d^2^/2, with D representing the longest diameter and d the shortest diameter.

### Cell lines, plasmids, reagents and transfection

A non-tumorigenic colon epithelial cell line CCD-112CoN (CCL1541) and colon cancer cell lines HCT116, RKO, Lovo, SW403, SW480, H508, SW1116, CT26, MC38, and SW1463 were purchased from the American Type Culture Collection (ATCC). Cells were cultured with DMEM or RPMI 1640 medium containing 10% FCS.

Transfection was performed using Lipofectamine 2000 reagent (Invitrogen) based on the manufacturer's instructions with miRNA mimics and siRNAs obtained from GenePharma.

Aiming to knockdown human SATB2, a gene-specific shRNA was cloned into the pLVX-shRNA1 plasmid purchased from Clontech, with a sequence targeting tGFP as the negative control. miR-449a overexpression was achieved using the pLVX-puro plasmid, which was also obtained from Clontech. Lentivirus was packaged using psPAX2 and pMD2G, and the three-plasmid system was used to obtain stable cell lines. The lentivirus supernatant was added to HCT116 or RKO cells, which were screened using 2 μg/ml puromycin for 2 weeks. To test for direct targeting of SATB2, Sirt1, and HDAC1 by miR-449a and miR-34a, we separately cloned their 3′UTRs into psi-CHECK^TM^2 (Promega); the primer sequences are listed in Supplementary Table S1. Mutation of the SATB2 3′UTR binding site for miR-449a was accomplished with a KOD-Plus-Mutagenesis Kit according to the manufacturer's instructions (Toyobo, SMK-101). Azacitidine (Aza) (Selleck S1782) was used at 10 μM; trichostatin A (TSA; Selleck S1045) was used as recommended at 330 nM.

### miRNA transfection

miRNA transfection was performed using miRNA mimics (GenePharma, Supplementary Table S1); NC served as the negative control.

### siRNA knockdown

Knockdown experiments for Sirt1, HDAC1, and DNMT3A were performed using siRNA oligonucleotides purchased from GenePharma. NC siRNA (GenePharma, Supplementary Table S1) was also transfected as the negative control.

### Luciferase reporter assay

HEK293 cells (ATCC) were cultured in DMEM; by 24 hours after seeding, the cells had grown to 70%–80% density. Using 1 μl Lipofectamine 2000, the cells were then transfected with 100 nM mimics (GenePharma) and 100 ng psi-CHECK2^TM^ containing either the wild-type or mutated 3′UTR of SATB2. After 24 hours, lysates were harvested, and reporter activity was measured in white-walled 96-well plates using the Dual-Luciferase Assay System (Promega). All experiments were performed in triplicate.

### RNA extraction and real-time PCR

Total RNA including miRNAs was isolated using TRIzol reagent (Invitrogen) according to the manufacturer's protocol. To obtain cDNA, Transcript First-Strand Synthesis Supermix (TransGen Biotech, AT301) was used to generate cDNA following the manufacturer's instructions using 1 μg RNA as the template. Actin was used as an internal control gene. For miRNA, 500 ng total RNA was reverse-transcribed into cDNA using a specific miRNA stem loop primer. miR-U6 was used as an internal control miRNA. qRT-PCR reagents purchased from TaKaRa and a 7900 Fast Real-Time PCR System (Applied Biosystems) were used for quantitative real-time reverse transcription (qRT-PCR). All samples were analyzed in triplicate. Error bars represent the standard deviation (SD), and statistical significance was calculated using a one-tailed, unpaired *t*-test. The relative quantification (RQ) was derived from the difference in cycle threshold (Ct) between the target gene and internal control (actin or miR-U6) compared to control cell lines using the formula RQ = 2^-ΔΔCt^. The mRNA and miRNA levels were assessed by SYBR Green–based quantitative real-time PCR with gene-specific primers (Supplementary Table S1).

### Western blot analysis

Harvested cells were washed with phosphate-buffered saline (PBS), and proteins were extracted using 1× loading lysis buffer. Equal amounts of protein, as measured by the Lowry protein assay, were separated by sodium dodecyl sulfate-polyacrylamide gel electrophoresis (SDS-PAGE) and transferred onto a polyvinylidenedifluoride (PVDF) membrane (Immobilon P, Millipore). The membrane was blocked in 5% non-fatty milk for 1 hour followed by immunoblotting with primary and horseradish peroxidase-conjugated secondary antibodies. Anti-SATB2 (SC-81376), anti-p27 (sc-528), and anti-actin (sc-1616) antibodies were purchased from Santa Cruz Biotechnology Inc. Anti-GAPDH monoclonal antibody was purchased from Kangcheng (KC-5G4).

Anti-Sirt1 (#8469) was purchased from Cell Signaling Technologies, and anti-HDAC1 (1G16) was purchased from Abmart Inc. Anti-DNMT3A was obtained from Dr. Xu Guoliang (Shanghai Institute of Biological Sciences, CAS). The proteins on the membranes were detected using SuperSignal West Pico Chemiluminescent Substrate (Pierce).

### WST-1 assay

HCT116 cells were seeded at a density of 1 × 10^3^ per well in 96-well plates and cultured at 37°C in a 5% CO_2_ incubator. Triple wells were seeded, and A450 was measured at 1, 2, 3, 4, and 5 days after seeding using a WST-1 Cell Proliferation and Cytotoxicity Assay Kit (Beyotime C0036) according to the manufacturer's protocol.

### Immunohistochemistry

Formalin-fixed, paraffin-embedded colorectal tumor tissue blocks from mice and from patients at Ruijin Hospital (Shanghai, China) were used in our investigation. Immunohistochemical staining was performed using primary antibodies and HRP-conjugated secondary antibodies. The antibodies used include anti-SATB2 (ab34735, Abcam), anti-p27 (sc-528, Santa Cruz Biotechnology Inc) and anti-Ki67 (sc-7846, Santa Cruz Biotechnology Inc). TUNEL staining was performed using an *In Situ* Cell Death Detection Kit (Roche) based on the manufacturer's instructions.

### Flow cytometry

In total, 1 × 10^5^ cells were seeded in a 6-well plate. After 48 hours, the cells were detached using 0.25% trypsin and washed. PE-Annexin V (BD Biosciences) and 7-AAD (BD Biosciences) were added for 30 minutes at 4°C in the dark before performing flow cytometry. The data were analyzed with FlowJo 7.6 software (TreeStar Inc).

### Statistical analysis

The cell growth curve A450 values, tumor volumes and weights and apoptosis-positive cell numbers are presented as the mean ± SD; the data were analyzed by Student's *t*-test. *p* < 0.05 was considered significant. Correlations between miR-449a and miR-34a were analyzed using SPSS (Statistical Product and Service Solutions).

## SUPPLEMENTARY MATERIALS FIGURES AND TABLES




