# Supplementary material for: miR-449a inhibits colorectal cancer progression by targeting SATB2
Source: Oncotarget. 2016 Jul 28;8(60):100975–88. doi: 10.18632/oncotarget.10900 (PMC5731849; doi:10.18632/oncotarget.10900)
Supplement: Supplementary file 1 [file oncotarget-08-100975-s001.pdf]

miR-449a inhibits colorectal cancer progression by targeting SATB2

Supplementary Materials

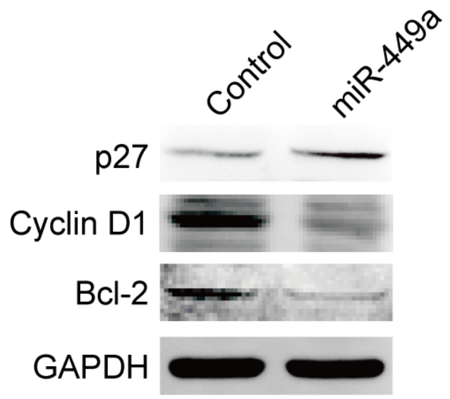

Supplementary Figure S1: miR-449a elevates the expression of p27 and reduces the expression of CyclinD1 and Bcl-2 in CRC cells. (**A**) miR-449a overexpression increased p27 and decreased Cyclin D1 and Bcl-2 protein level in HCT116 cells.

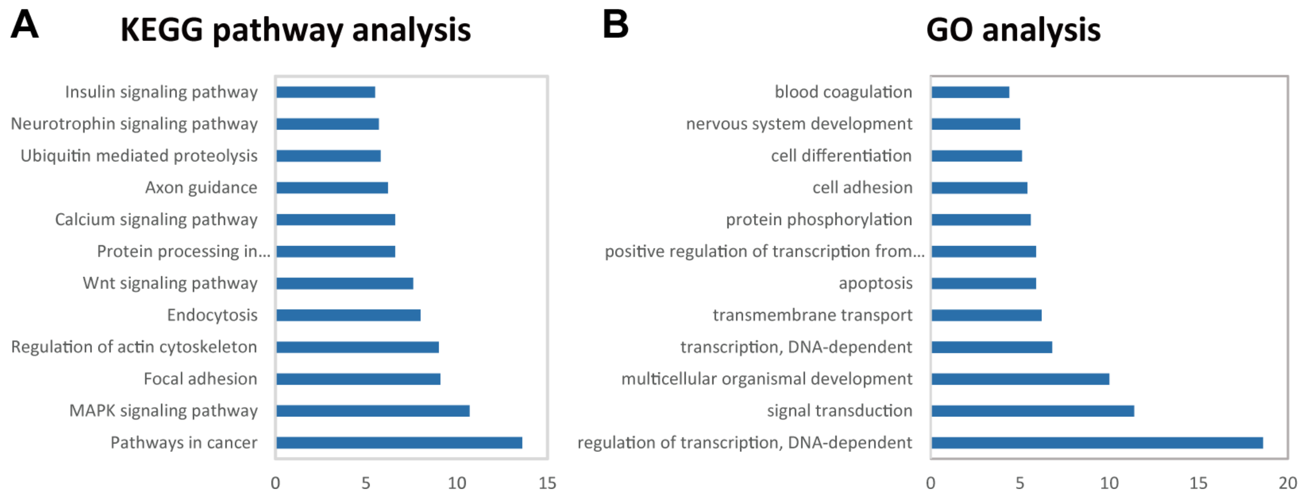

Supplementary Figure S2: AZA and TSA treatment reverses a series of pathways and functions. (**A**) KEGG pathway analysis after AZA and TSA treatment in RKO cells. (**B**) GO analysis after AZA and TSA treatment in RKO cells.

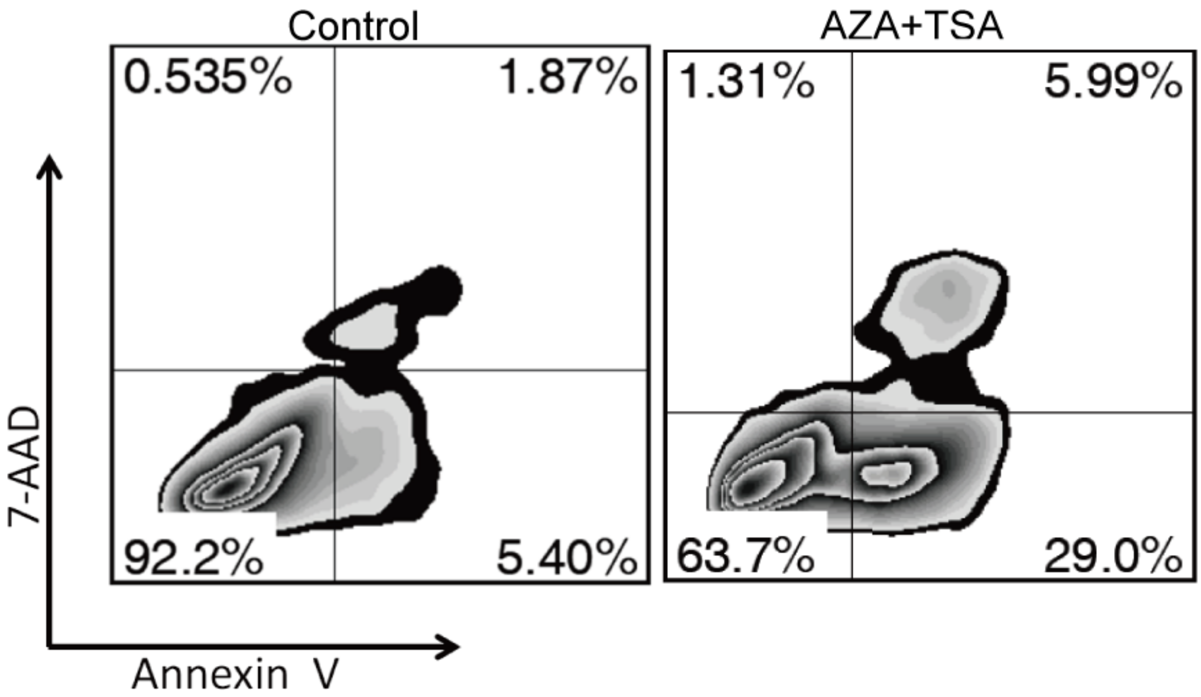

Supplementary Figure S3: AZA and TSA treatment induces apoptosis. Annexin V/7-AAD flow cytometry results of HCT116 cells after AZA and TSA treatment.

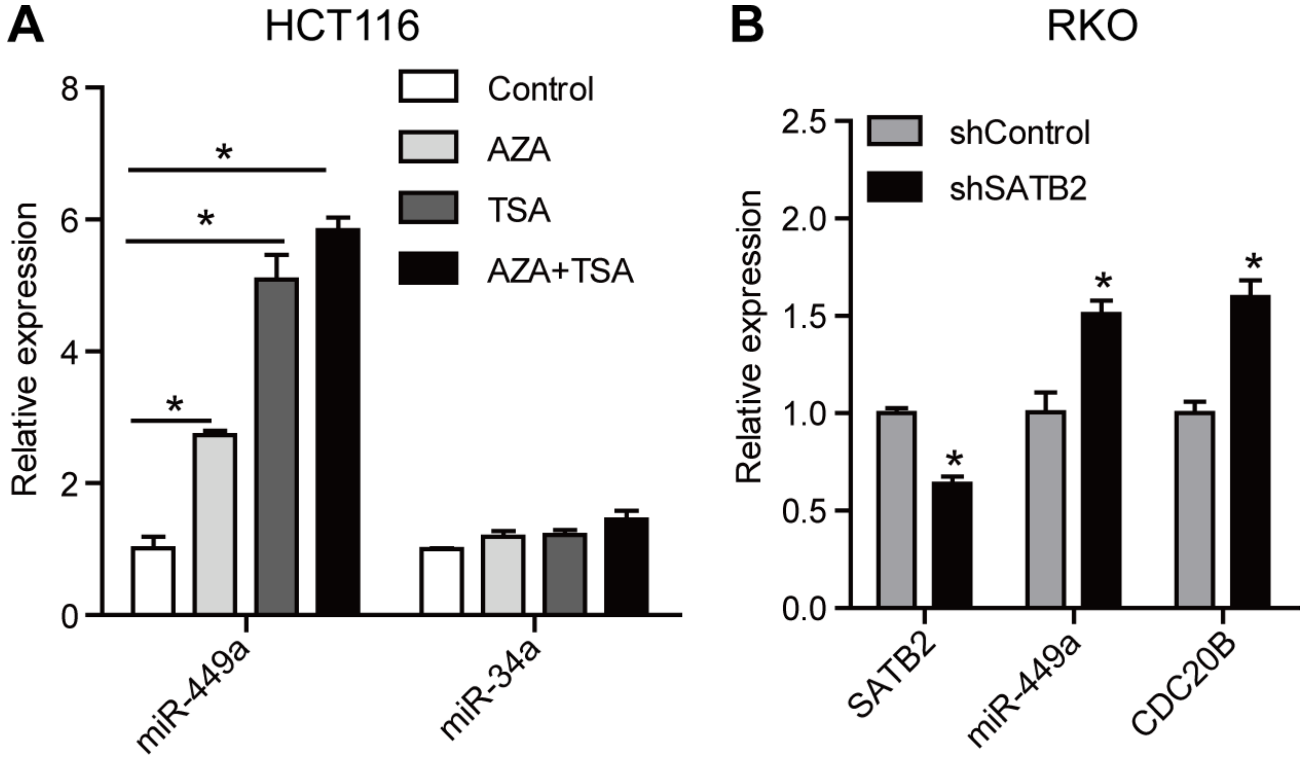

Supplementary Figure S4: miR-449a is activated by AZA and TSA treatment and SATB2 knockdown. (**A**) miR-449a and miR-34a mRNA levels after AZA, TSA or AZA + TSA treatment in HCT116. (**B**) SATB2, miR-449a, and CDC20B mRNA levels after SATB2 knockdown in RKO cells.

Supplementary Table S1: Primers or sequences for construction of plasmids, real-time, microRNA mimics or siRNAs. See Supplementary_Table_S1

Supplementary Table S2: The clinic pathogenic characteristics and clinical pathological stages of 50 patients

No.	Age	Gender	Stage	Death	Tumor differentiation
1	55	female	I	no	medium
2	50	female	I	no	medium
3	62	female	I	no	medium
4	75	male	I	no	high
5	26	female	I	no	medium
6	77	male	I	no	medium
7	72	male	I	no	low
8	50	male	I	no	medium
9	40	female	I	no	low
10	70	female	I	no	medium
11	59	male	II	yes	medium
12	80	male	II	no	medium
13	55	male	II	no	medium
14	77	male	II	no	medium
15	67	male	II	no	medium
16	65	male	II	no	medium
17	71	female	II	no	low
18	61	male	II	no	medium
19	51	male	II	no	medium
20	55	female	IIA	yes	medium
21	89	male	IIA	yes	medium
22	74	female	IIA	yes	low
23	77	male	IIA	yes	medium
24	70	female	IIB	no	medium
25	53	male	IIB	no	medium
26	69	female	IIIC	no	medium
27	52	male	IIIC	no	medium
28	61	male	IIIC	no	medium
29	63	male	IIIC	no	medium
30	70	female	IIIA	no	medium
31	54	male	IIIA	no	medium
32	65	male	IIIB	no	medium
33	62	male	IIIB	no	low
34	75	female	IIIB	no	low
35	62	female	IIIC	yes	low
36	76	male	IVA	yes	medium
37	54	male	IVA	no	low
38	50	male	IVA	no	medium
39	70	male	IVA	no	low
40	78	male	IVA	yes	low
41	67	female	IVA	no	medium
42	70	male	IVB	yes	medium
43	58	male	IVB	yes	medium
44	42	male	IVB	yes	medium
45	72	female	IVB	yes	low
46	63	male	IVA	yes	medium
47	75	female	IVA	yes	medium
48	77	female	IVB	yes	medium
49	69	female	IVB	yes	low
50	71	female	IVB	yes	low
