# Supplementary material for: miR-449a inhibits colorectal cancer progression by targeting SATB2
Source: Oncotarget. 2016 Jul 28;8(60):100975–88. doi: 10.18632/oncotarget.10900 (PMC5731849; doi:10.18632/oncotarget.10900)
Supplement: Supplementary file 2 [file oncotarget-08-100975-s002.docx]

| Name | Plasmid |  | Primers 5’-3’/Sequence 5’-3’ | |
| --- | --- | --- | --- | --- |
| SATB2 CDS | pcDNA3.1(+) | EcoRI | Forward | CGGAATTCATGGAGCGGCGGAGCGAGAG |
|  |  | XbaI | Reverse | GCTCTAGATTATCTCTGGTCAATTTCGGC |
| hsa-miR-449a | pLVX-puro | EcoRI | Forward | ATAGAATTCGATTAGAGTTCTGCCACTATG |
|  |  | XbaI | Reverse | ATATCTAGAAAGTAATTGTAATACTTTCC |
| shSATB2-1 | pLVX-shRNA1 | BamHI | Forward | gatccGCGACATGCTACAAGATGTCTATTCAAGAGATAGACATCTTGTAGCATGTCGTTTTTTg |
|  |  | EcoRI | Reverse | aattcAAAAAACGACATGCTACAAGATGTCTATCTCTTGAATAGACATCTTGTAGCATGTCGCg |
| shSATB2-2 | pLVX-shRNA1 | BamHI | Forward | gatccGCCATGCAGAATTTCCTCAATTTCAAGAGAATTGAGGAAATTCTGCATGGCTTTTTTg |
|  |  | EcoRI | Reverse | aattcAAAAAAGCCATGCAGAATTTCCTCAATTCTCTTGAAATTGAGGAAATTCTGCATGGCg |
| SATB2 3’UTR-1 | psi-CHECK2 | XhoI | Forward | ATACTCGAGttgcaatccgaaagaata |
|  |  | NotI | Reverse | ATAGCGGCCGCtgatcaagacttgcacta |
| SATB2 3’UTR-2 | psi-CHECK2 | XhoI | Forward | ATACTCGAGctgtagtatgctgcagctt |
|  |  | NotI | Reverse | ATAGCGGCCGCgtcaagaggcactacaagag |
| SATB2 3’UTR-1  mutant | psi-CHECK2 |  | Forward | caaaataaacgacaccagtgaaaaaaaaaaaagt |
|  |  |  | Reverse | acttttttttttttcactggtgtcgtttattttg |
| SATB2 3’UTR-2  mutant | psi-CHECK2 |  | Forward | attttttaatttcaccagtaagtttgcagtggtt |
|  |  |  | Reverse | aaccactgcaaacttactggtgaaattaaaaaat |
| SATB2 3’UTR | psi-CHECK2 | XhoI | Forward | ATACTCGAGttgcaatccgaaagaata |
|  |  | NotI | Reverse | ATAGCGGCCGCgtcaagaggcactacaagag |
| Sirt1 3’UTR | psi-CHECK2 | XhoI | Forward |  |
|  |  | NotI | Reverse |  |
| HDAC1 3’UTR | psi-CHECK2 | XhoI | Forward |  |
|  |  | NotI | Reverse |  |
| U6 RT |  |  |  | GTCGTATCCAGTGCAGGGTCCGAGGTATTCGCACTGGATACGACAAAATA |
| hsa-miR-449a RT |  |  |  | GTCGTATCCAGTGCAGGGTCCGAGGTATTCGCACTGGATACGACACCAGC |
| hsa-miR-34a RT |  |  |  | GTCGTATCCAGTGCAGGGTCCGAGGTATTCGCACTGGATACGACACAACC |
| U6 real time PCR |  |  | Forward | CGGCGGTCGTGAAGCGTTCCAT |
|  |  |  | Reverse | CCAGTGCAGGGTCCGAGGTAT |
| hsa-miR-449a real time PCR |  |  | Forward | CGGCGGTTGGCAGTGTATTGTTA |
|  |  |  | Reverse | CCAGTGCAGGGTCCGAGGTAT |
| hsa-miR-34a real time PCR |  |  | Forward | CGGCGGTTGGCAGTGTCTTAGCT |
|  |  |  | Reverse | CCAGTGCAGGGTCCGAGGTAT |
| actin real time PCR |  |  | Forward | CTGGAACGGTGAAGGTGACA |
|  |  |  | Reverse | AAGGGACTTCCTGTAACAATGCA |
| CDC20B real time PCR |  |  | Forward | ACTCGCAACAGGAACCTCTG |
|  |  |  | Reverse | GAAGTCAAGATTCCGCCAAC |
| SATB2 real time PCR |  |  | Forward | CAAAGAATGCCCTCTCTC |
|  |  |  | Reverse | CATGATGGGCTGTAATGC |
| DNMT3A real time PCR |  |  | Forward | CCGATGCTGGGGACAAGAAT |
|  |  |  | Reverse | CCCGTCATCCACCAAGACAC |
| Sirt1 real time PCR |  |  | Forward | TAGCCTTGTCAGATAAGGAAGGA |
|  |  |  | Reverse | ACAGCTTCACAGTCAACTTTGT |
| HDAC1 real time PCR |  |  | Forward | CATCTCCTCAGCATTGGCTT |
|  |  |  | Reverse | CGAATCCGCATGACTCATAA |
| NC |  |  | Sense | UUCUCCGAACGUGUCACGUdTdT |
|  |  |  | Anitsense | ACGUGACACGUUCGGAGAAdTdT |
| human miR-449a mimics |  |  | Sense | UGGCAGUGUAUUGUUAGCUGGU |
|  |  |  | Anitsense | CAGCUAACAAUACACUGCAAUU |
| human miR-34a mimics |  |  | Sense | UGGCAGUGUCUUAGCUGGUUGU |
|  |  |  | Anitsense | AACCAGCUAAGACACUGCAAUU |
| anti-miR-449a |  |  | ACCAGCUAACAAUACACUGCCA | |
| human Sirt1 siRNA |  |  | GAAGUUGACCUCCUCAUUGU | |
| human HDAC1 siRNA |  |  | CAGCGACUGUUUGAGAACC | |
| human DNMT3A siRNA |  |  | CCAGAUGUUCUUCGCUAAU | |
